# Novel insights into the association between organ damage and inflammatory response in preoperative abdominal aortic aneurysms

**DOI:** 10.3389/fcvm.2025.1511112

**Published:** 2025-04-24

**Authors:** Huan Wen, Bo Su, Jinbo Liu, Hongyu Wang

**Affiliations:** ^1^ Department of Vascular Medicine, Peking University Shougang Hospital, Beijing, China; ^2^ Beijing Shijingshan District Key Clinical Specialty of Vascular Medicine, Beijing, China; ^3^ Institute of Clinical Pharmacology, Peking University First Hospital, Beijing, China; ^4^ Center for Vascular Health Research, Peking University School of Medicine, Beijing, China; ^5^ National Key Laboratory of Vascular Homeostasis and Remodeling, Peking University, Beijing, China; ^6^ Center for Heart and Vascular Health, Peking University Clinical Research Institute, Beijing, China; ^7^ Heart and Vascular Health Research Center of Chengdu Medical College (HVHRC-CMC), Chengdu, China; ^8^ Intelligent Heart and Vascular Health Digital Management Research Center, National Institute of Health Data Science At Peking University, Beijing, China

**Keywords:** abdominal aortic aneurysm, inflammation, blood urea nitrogen, creatinine, kidney injury

## Abstract

**Background:**

Abdominal aortic aneurysm (AAA) is a life-threatening condition in the elderly population. The insidious nature of AAA onset makes early detection difficult. Currently, there are few studies on changes in laboratory parameters during AAA development.

**Methods:**

This study included 55 elderly patients with AAA who were admitted to the Department of Vascular Medicine, Shougang Hospital, Peking University 2021–2022. Propensity score matching (PSM) in a 1:1 ratio was performed to match the 55 patients and 1,031 controls. In this population of AAA, correlation and regression analyses were used to explore the association between the level of inflammation and each laboratory parameter.

**Results:**

Compared to the control group, significant differences in inflammatory markers, transaminase and bilirubin levels, blood urea nitrogen (BUN) and creatinine (Cr) levels, and ankle-brachial index were found in the aneurysm group. After PSM, the differences between the two groups for each parameter remained statistically significant. Correlation and regression analyses showed a weak positive correlation between the inflammatory index and the BUN and Cr levels (correlation coefficient = 0.22).

**Conclusions:**

Our study demonstrates the presence of a highly inflammatory state and damage to various organs in patients with AAA. This hyperinflammatory state may be associated with kidney injury and is a cause of concern.

## Introduction

Abdominal aortic aneurysm (AAA) is a serious vascular disease with a prevalence that increases with age and is particularly significant in the elderly population (1, 2). The incidence of AAA is higher in males than in females, and tends to increase annually (3, 4). Smoking, hypertension, coronary heart disease (CHD), and genetics are known risk factors for AAA (5, 6). An aneurysm is formed when the abdominal aorta undergoes permanent restrictive dilatation with an increase in vessel diameter greater than 50%, and the larger the diameter of the aneurysm, the greater the risk of rupture (2, 7). AAA has no obvious symptoms in the early stages, but pain, nausea, and vomiting may occur when the aneurysm increases in size (8, 9). The diagnosis of AAA is dependent on imaging, and early detection and diagnosis are key to its treatment (10).

Inflammation is the defensive response of the body to stimuli (11, 12). Inflammation plays an important role in the development of cardiovascular disease (13). Laboratory tests, including routine blood tests, C-reactive protein, blood sedimentation, and other inflammatory indicators, can assess the inflammatory status of the body (14). When the body releases large amounts of inflammatory mediators and cytokines, this hyperinflammatory state triggers a series of clinical signs and symptoms (15). Previous studies have shown that a more severe preoperative inflammatory response in patients with AAA can lead to an increase in postoperative complications, thereby affecting patient prognosis (16, 17). However, although studies have focused on the relationship between preoperative inflammatory response and prognosis in AAA, there is still a relative lack of studies on the association between preoperative inflammatory response and changes in organ function in AAA. This study aimed to investigate the relationship between preoperative inflammatory markers of AAA and various laboratory parameters and to assess functional changes in the organ.

## Methods

### Data source and study design

The population for this analysis was derived from patients with AAA admitted to the Department of Vascular Medicine at Shougang Hospital of Peking University, and the diagnosis of AAA was confirmed after rigorous imaging evaluation. AAA was diagnosed in patients with more than 50% increase in abdominal aortic diameter on the imaging report, and 55 patients who came to the hospital from February 2021 to December 2022 were finally included. More than 90% of these patients had taken antihypertensive and lipid-lowering medications prior to diagnosis. The control population for AAA, also from this vascular department, was selected from those who came to the clinic between 2011 and 2015 but whose imaging assessment was not an aneurysm, and a total of 1,031 were included. The Ethics Committee of Shougang Hospital of Peking University approved the study protocol. All the participants provided written informed consent.

### Variable collection

The medical registration of patients with AAA and the control population was verified by two physicians to collect information about the participants' sex, age, smoking habits, and previous illnesses. Current smoking was defined as having smoked > 100 cigarettes in a lifetime and currently still smoked daily (18). The diagnosis of previous diseases, such as hypertension, diabetes, and CHD, depended on the participant being told by a doctor or being on relevant medication themselves (19).

Anthropometric measurements were performed by physicians in the department. Body mass index (BMI) was determined by dividing the participant's weight in kilograms by the square of height in meters. Brachial and ankle artery systolic blood pressure (SBP) measurements were performed for each participant using a mercury sphygmomanometer, and the ankle-brachial index (ABI) was determined by dividing the highest ankle artery SBP by the highest brachial artery SBP. Before performing the measurements, the participants were asked to rest in the supine position for at least 10 min while ABI was measured on the right and left sides. The ABI of the participants in the study was calculated as the mean of the left and right sides. ABI reflects the peripheral vascular function (20).

After overnight fasting, venous blood samples were collected from the participants to measure various biochemical markers, including C-reactive protein (CRP), alanine transaminase (ALT), aspartate transaminase (AST), blood urea nitrogen (BUN), creatinine (Cr), total bilirubin (TBil), and direct bilirubin (DBil). CRP, ALT, AST, BUN, Cr, TBil, and DBil levels were measured in the laboratory of the Shougang Hospital of Peking University using standard methods. ALT, AST, TBil, and DBil levels reflect liver function. BUN and Cr levels reflect renal function. The venous blood was divided into different tubes according to the requirements of the laboratory, and then tested using a fully automated biochemical analyzer (Hitachi 7,170) manufactured by Hitachi, Tokyo, Japan, based on the principle of spectrophotometry. Platelet, neutrophil, and lymphocyte counts were obtained from all participants. Systemic immune inflammatory index SII = platelet count (×10^−9^/L) × absolute neutrophil count (×10^−9^/L)/absolute lymphocyte count (×10^−9^/L) (21). CRP and SII levels reflect the systemic inflammatory status of the participants (22).

### Statistical analysis

Data processing and statistical analyses were performed using IBM SPSS version 27 and R software version 4.3.2. Plotting was performed using the R software and GraphPad Prism version 9.5.1. Categorical variables were expressed as frequencies and percentages. Continuous variables showing a normal distribution were expressed as mean ± standard deviation, while continuous variables showing a skewed distribution were expressed as medians and interquartile ranges. Because SII showed a skewed distribution, all SII values were converted to logarithmic values with a base of 10 and expressed using LogSII, which is normally distributed. The chi-square test was used to compare categorical variables. In addition, the independent t-test and Mann–Whitney test were used to compare normally and non-normally distributed continuous variables, respectively.

In the present study, propensity score matching (PSM) was performed in a 1:1 ratio without replacement using the MatchIt R software package (version 11). Matching was performed using the nearest-neighbor principle, which matches patients with aneurysm and control populations based on propensity scores calculated from a combination of variables. This rigorous approach ensured that the case and control groups were effectively balanced in terms of their propensity scores. Variables used to calculate the propensity score included sex, age, BMI, smoking history, and history of previous disease (23), thus accounting for key factors that may have influenced the results of the study.

After PSM, histograms and densitograms were used to compare the levels of inflammation between the case and control groups. Correlation and regression analyses were used to analyze the association between inflammatory indicators and each laboratory parameter in the case group. Statistical *p*-value < 0.05.

## Results

### Characteristics of the study population

The clinical and laboratory characteristics of the AAA and control groups are shown in Table 1. 55 patients had a mean age of 75.0 ± 8.2 years, whereas 1,031 controls had a mean age of 64.2 ± 12.1 years, and the proportion of males was higher in the case group than in the control group. This is consistent with epidemiological data on AAA. History of smoking and hypertension was statistically different between the two groups. CRP and SII levels were compared between the two groups, and inflammation levels were significantly higher in the case group than in the control group. There were statistically significant differences in ALT, AST, BUN, Cr, TBil, DBil levels, and ABI. Compared with the control group, the case group may have impaired liver, kidney, and vascular functions.

**Table 1:** Basic characteristics of the study population according to state of disease.

Variables	Aneurysm (*N* = 55)	Control (*N* = 1,031)	*P*-value
Age, years	75.0 ± 8.2	64.2 ± 12.1	<0.001
Men, *n* (%)	42 (76.4)	468 (45.4)	<0.001
BMI, kg/m^2^	24.5 ± 3.3	25.3 ± 3.5	0.081
Current smoking, *n* (%)	35 (63.6)	154 (14.9)	<0.001
Hypertension, *n* (%)	48 (87.3)	672 (65.2)	<0.001
DM, *n* (%)	18 (32.7)	289 (28.0)	0.451
CHD, *n* (%)	31 (56.4)	438 (42.5)	0.043
CRP, mg/L	15.1 (5.4, 27.8)	1.7 (0.8, 3.5)	<0.001
Neutrophils, ×10^9^/L	7.9 ± 2.5	3.9 ± 1.5	<0.001
Lymphocytes, ×10^9^/L	1.4 ± 0.3	1.7 ± 0.6	<0.001
Platelet, ×10^9^/L	222.3 ± 42.5	201.8 ± 52.4	0.004
SII, ×10^9^/L	1197.0 (911.8, 1749.4)	444.5 (305.1, 646.0)	<0.001
LogSII	3.1 ± 0.2	2.7 ± 0.3	<0.001
ALT, U/L	30.9 ± 15.7	21.0 ± 12.7	<0.001
AST, U/L	27.5 ± 11.4	24.4 ± 8.4	0.009
BUN, mmol/L	7.0 ± 1.8	5.5 ± 1.8	<0.001
Cr, µmol/L	103.1 ± 35.7	69.3 ± 22.2	<0.001
TBil, mmol/L	15.0 ± 4.7	13.3 ± 4.8	0.010
DBil, mmol/L	5.2 ± 1.7	3.8 ± 1.6	<0.001
R-ABI	0.9 ± 0.2	1.1 ± 0.2	<0.001
L-ABI	0.9 ± 0.2	1.1 ± 0.2	<0.001

The values in the table are presented as the mean ± standard deviation, median (interquartile range), or *n* (%). BMI, body mass index; DM, diabetes mellitus; CHD, coronary heart disease; CRP, C-reactive protein; SII, systemic immune-inflammation index; ALT, alanine aminotransferase; AST, aspartate aminotransferase; BUN, blood urea nitrogen; Cr, creatinine; TBil, total bilirubin; DBil, direct bilirubin; R-ABI, right ankle brachial index; L-ABI, left ankle brachial index.

### Propensity-matched analysis

**Figure 1:**
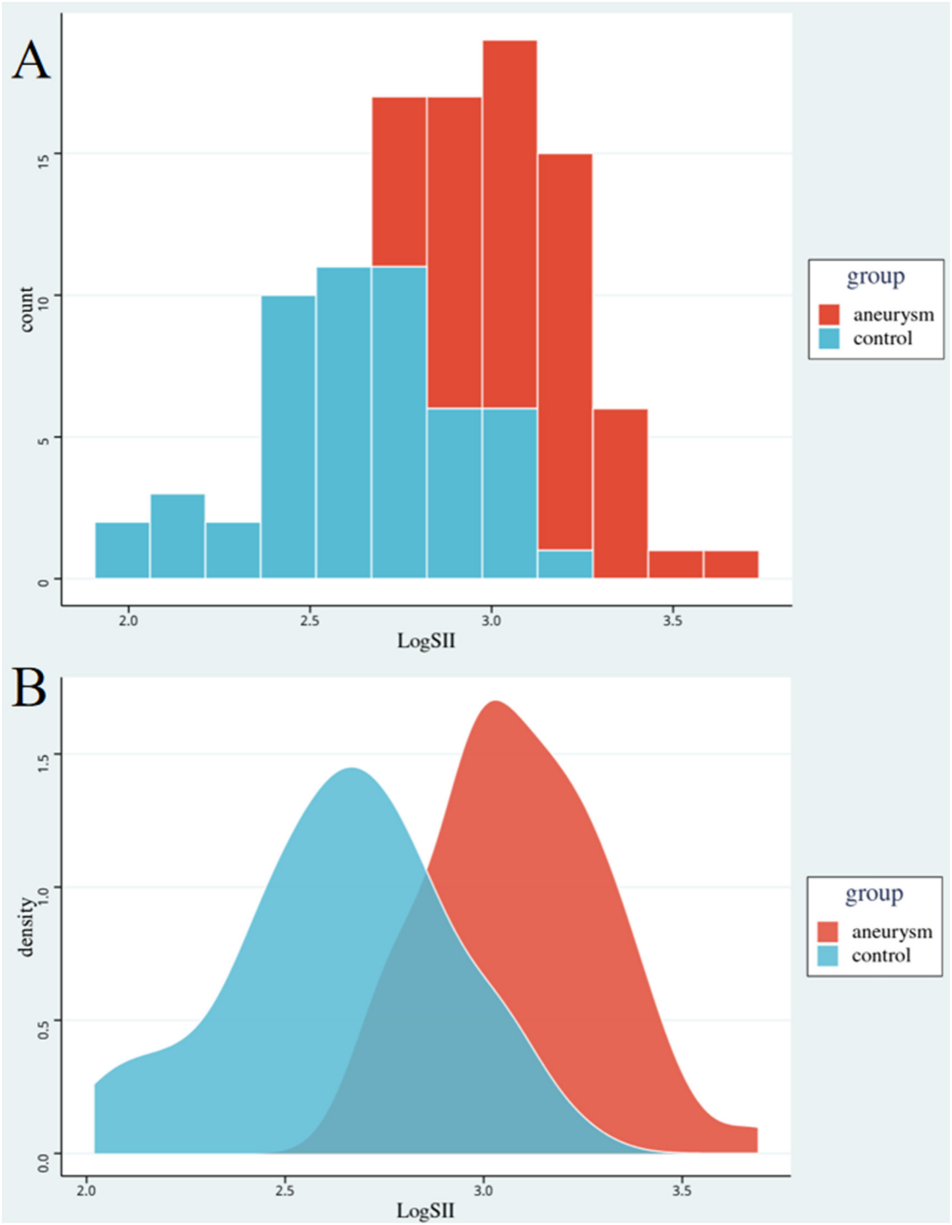
Comparison of inflammation levels between the two groups. Red represents the abdominal aortic aneurysm group, blue represents the control group. Graph **A** is a histogram of the level of inflammation. Graph **B** is a density map of the level of inflammation. The horizontal axis is the logarithm of SII. SII, systemic immune-inflammation index.

Table 2 shows the clinical and laboratory characteristics of the AAA and control groups after the PSM. The mean age of the 52 patients was 74.8 ± 8.2 years, whereas that of the 52 controls was 73.7 ± 9.6 years. Statistically insignificant differences in sex ratio, smoking history, and history of hypertension were observed in the case group compared with the control group, which in turn attenuated the interference of relevant factors in the comparison of inflammation levels between the two groups. Comparison of CRP and SII levels between the two groups showed that the inflammation level in the case group remained significantly higher than that in the control group. Statistically significant differences were found in ALT, AST, Cr, and DBil levels, and ABI. Statistical differences in BUN and TBil levels were attenuated in both the groups. Figure 1 shows LogSII to represent the level of inflammation, demonstrating the hyperinflammatory state of the patient group.

**Table 2:** Basic characteristics of the study population according to state of disease after propensity score matching (1:1).

Variables	Aneurysm (*N* = 52)	Control (*N* = 52)	*P*-value
Age, years	74.8 ± 8.2	73.7 ± 9.6	0.511
Men, *n* (%)	39 (75.0)	37 (71.2)	0.658
BMI, kg/m^2^	24.5 ± 3.4	24.0 ± 2.9	0.469
Current smoking, *n* (%)	32 (61.5)	34 (65.4)	0.684
Hypertension, *n* (%)	45 (86.5)	45 (86.5)	1.000
DM, *n* (%)	16 (30.8)	17 (32.7)	0.833
CHD, *n* (%)	29 (55.8)	29 (55.8)	1.000
CRP, mg/L	15.4 (5.5, 28.3)	1.9 (1.2, 3.6)	<0.001
Neutrophils, ×10^9^/L	8.0 ± 2.5	4.1 ± 1.5	<0.001
Lymphocytes, ×10^9^/L	1.4 ± 0.3	1.7 ± 0.6	0.007
Platelet, ×10^9^/L	221.4 ± 43.2	188.1 ± 49.0	<0.001
SII, ×10^9^/L	1195.9 (913.5, 1743.6)	448.8 (304.5, 655.1)	<0.001
LogSII	3.1 ± 0.2	2.6 ± 0.3	<0.001
ALT, U/L	30.3 ± 15.5	17.6 ± 10.8	<0.001
AST, U/L	27.1 ± 11.4	22.7 ± 6.4	0.016
BUN, mmol/L	7.0 ± 1.9	6.5 ± 2.9	0.243
Cr, µmol/L	104.2 ± 36.4	84.1 ± 37.2	0.006
TBil, mmol/L	14.9 ± 4.7	13.9 ± 5.2	0.334
DBil, mmol/L	5.1 ± 1.7	4.2 ± 1.7	0.008
R-ABI	0.9 ± 0.2	1.0 ± 0.2	0.024
L-ABI	0.9 ± 0.2	1.0 ± 0.2	0.034

The values in the table are presented as the mean ± standard deviation, median (interquartile range), or *n* (%). BMI, body mass index; DM, diabetes mellitus; CHD, coronary heart disease; CRP, C-reactive protein; SII, systemic immune-inflammation index; ALT, alanine aminotransferase; AST, aspartate aminotransferase; BUN, blood urea nitrogen; Cr, creatinine; TBil, total bilirubin; DBil, direct bilirubin; R-ABI, right ankle brachial index; L-ABI, left ankle brachial index.

### Correlation and regression analysis

**Figure 2:**
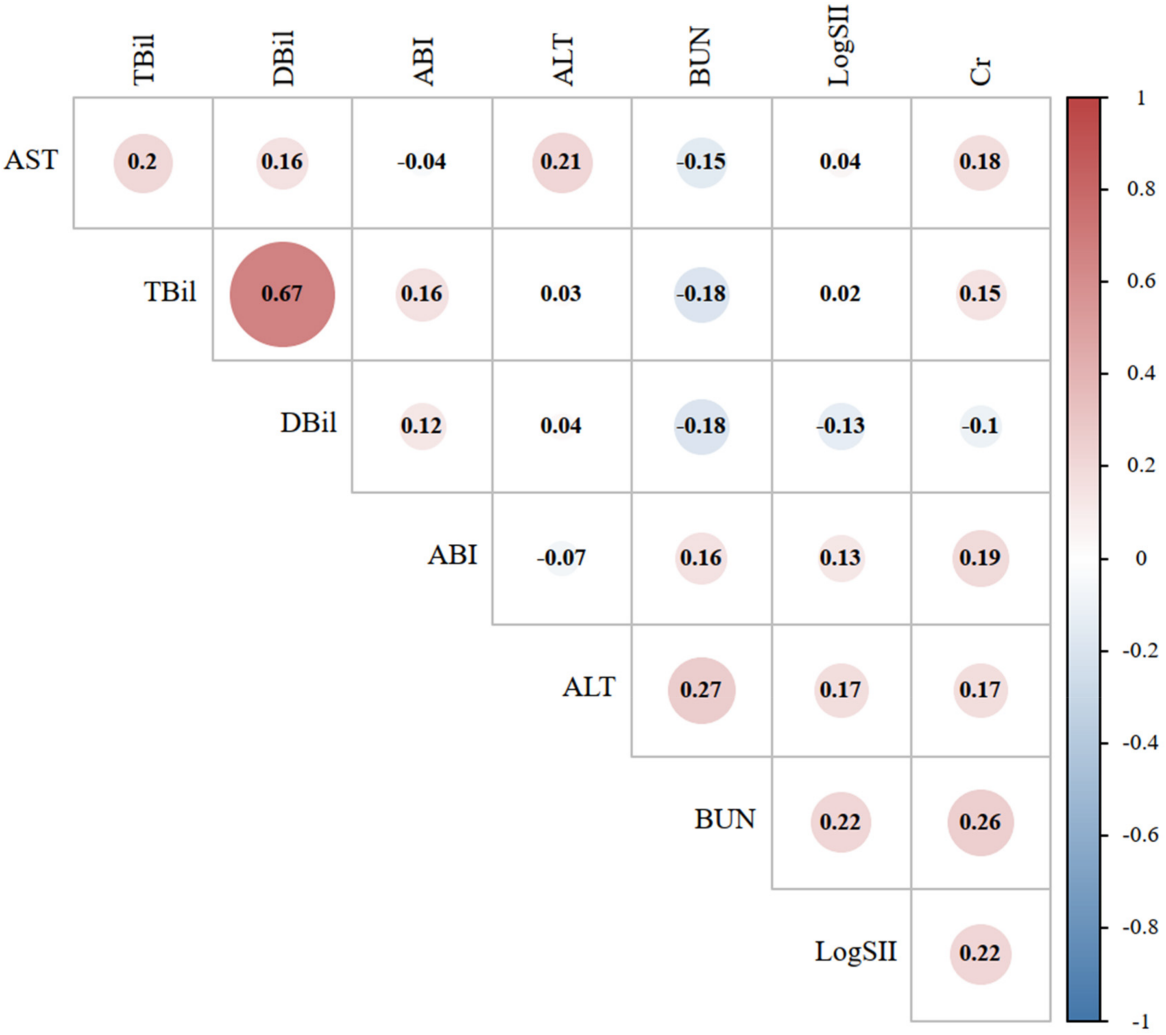
Correlation graph between the laboratory indicators in the abdominal aortic aneurysm group. The size of the circle represents the strength of the correlation. Red represents a positive correlation, while blue represents a negative correlation. SII, systemic immune-inflammation index; ALT, alanine aminotransferase; AST, aspartate aminotransferase; BUN, blood urea nitrogen; Cr, creatinine; TBil, total bilirubin; DBil, direct bilirubin.

**Figure 3:**
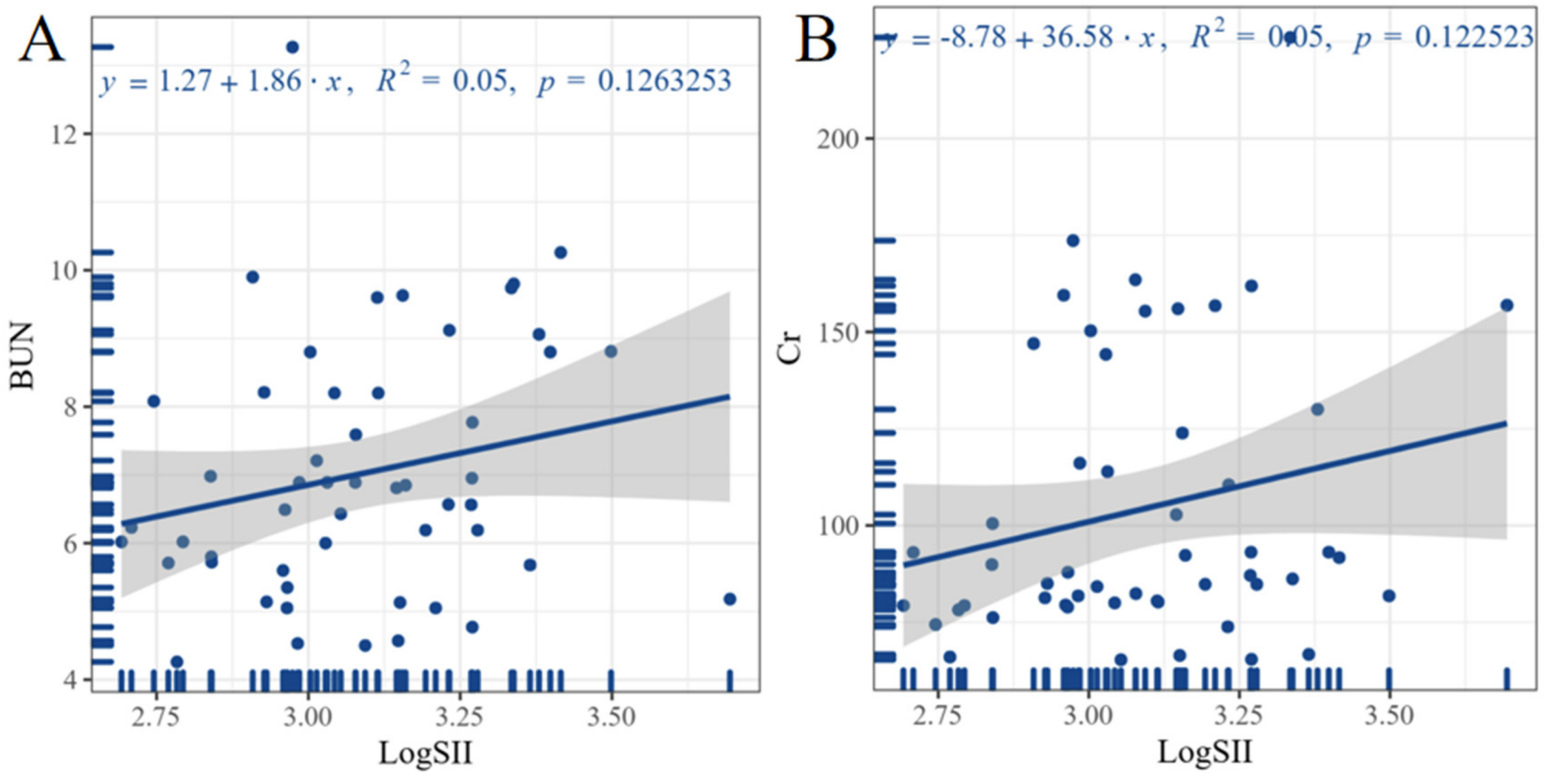
Linear regression analysis between inflammation levels and laboratory indicators. Graph **A** illustrates the linear regression relationship between LogSII and BUN. Graph **B** illustrates the linear regression relationship between LogSII and Cr. The equation for linear regression is shown at the top of the image. SII, systemic immune-inflammation index; BUN, blood urea nitrogen; Cr, creatinine.

Figure 2 shows the correlation analysis between LogSII and ALT, AST, BUN, Cr, TBil, DBil, and ABI in the case group. The highest correlation was found between LogSII and BUN and Cr levels, with a correlation coefficient of 0.22, but the correlation remained weak. Linear regression can further quantify the trend between the two variables. Figure 3 shows the linear regression between LogSII and BUN and Cr; as LogSII increased, BUN and Cr both increased to varying degrees.

## Discussion

Our study compared the laboratory parameters between the AAA and control groups. Inflammation, transaminase, bilirubin, and Cr levels were significantly higher in the case group than in the control group, and these indices were statistically significant between the two groups before and after PSM. There was a positive correlation between inflammation level and renal function evaluation parameters in the case group, and it is possible that there was an increased risk of kidney injury in the high inflammatory state. However, linear regression analysis of the level of inflammation and renal function evaluation parameters in the case group showed a non-significant relationship, which may be related to the state of AAA where the renal blood supply is affected, among other things.

To the best of our knowledge, this study is the first to investigate the association between preoperative inflammatory status and organ damage in AAA patients. PSM was also used to attenuate the interference of confounding factors between the case and control groups. Few clinical studies have been conducted on AAA. In a study of 79 patients with AAA who underwent endovascular aneurysm repair, Shalaby SY et al. found that patients with preoperative systemic inflammation experienced more postoperative complications (17). Windsor et al. selected 20 patients with AAA and 20 controls for their study and found that a higher intensity of exercise induced a decrease in inflammatory factors in patients with AAA, such as matrix metalloproteinase-9 (MMP-9) and tumor necrosis factor alpha (TNF-α) (24). Golledge J et al. collected data on 997 elderly male patients with AAA and found that the patients had higher levels of the inflammatory mediator angiopoietin-2, and that increased angiopoietin-2 was associated with increased risk of cardiovascular death (25). In a retrospective study of 373 patients undergoing elective endovascular aortic aneurysm repair, King AH et al. found that patients with an elevated preoperative neutrophil-lymphocyte ratio (NLR) had an increased risk of postoperative mortality (26). A survey of 99 postoperative AAA patients by Muehling et al. found that perioperative advanced nursing care significantly reduced the rates of systemic inflammatory response syndrome (SIRS) and organ failure (27). Our study found that most patients with AAA had a history of hypertension and were on antihypertensive and lipid-lowering medications, and therefore rational control of blood pressure may be beneficial to the stabilization of the patient's condition.

Inflammation is a central factor in the development of AAA (28). Primary inflammation of the vessel wall leads to progressive dilatation of the abdominal aorta (29). After the formation of an AAA, the inflammatory response may be further exacerbated by a series of pathological changes that may occur in the surrounding tissues and vessel walls, such as thickening of the vessel wall, fibrosis, and infiltration of inflammatory cells (30). AAA is a local autoimmune response, and complement activation amplifies the inflammatory response (31). When lipids in the aneurysmal plaque leak into the surrounding tissues, they act as allergens that trigger an immune response, leading to inflammation (32). In addition, embolization of aortic trophoblastic vessels may trigger intima-media damage, accompanied by an inflammatory response (33). Lymphatic stasis and retroperitoneal edema due to lymphatic vessel compression also play roles in the inflammatory response (34). In patients with AAA, the inflammatory response is exacerbated by a history of smoking and hypertension (35).

Inflammation is the body's defense response to external stimuli, which is beneficial to the body; however, the function of all organs in the body is damaged in a state of high inflammation (36). In a state of high inflammation, a large number of inflammatory and anti-inflammatory factors are distributed to tissues and organs throughout the body via blood circulation (37). These factors act on the capillary wall, leading to damage to capillary endothelial cells and resulting in microcirculation disorders (38). Microstructural changes occur in organs, such as thickening of the vessel wall, narrowing of the lumen, and fibrosis. These changes, in turn, affect the normal function of the organs (37, 38). Kidney damage results in decreased urine output and increased blood Cr levels (39). Liver damage causes jaundice and elevated transaminase levels (40). Therefore, preoperative monitoring of the patient's inflammation level may help to protect the organs, and the development of a timely and dynamic monitoring device in the future will be more useful for the preoperative preparation of the patient.

This study has several limitations. First, as this was a cross-sectional survey study, it was not possible to clarify the relationship between dynamic changes in inflammation levels and organ damage. Second, although the interference of relevant confounders, such as smoking history and history of previous diseases, was attenuated by PSM, other confounders, such as drug use, could not be avoided. Third, the values of transaminase, bilirubin, BUN, and Cr used in our assessment of hepatic and renal function were based on the results on the day of the study, which may be confounded by a variety of factors. Fourth, the population we studied was single-center and had a limited sample size, and we did not investigate multicenter data, and the results may have been limited in extensibility.

## Conclusions

In conclusion, patients with AAA have a hyperinflammatory state preoperatively, and a hyperinflammatory response may be associated with organ damage. Therefore, preoperative monitoring and attention to inflammatory markers and changes in organ function are advocated.

## Data Availability

The raw data supporting the conclusions of this article will be made available by the authors, without undue reservation.

## References

[1] Harrison SC Holmes MV Burgess S Asselbergs FW Jones GT Baas AF Genetic association of lipids and lipid drug targets with abdominal aortic aneurysm: a meta-analysis. JAMA Cardiol. (2018) 3:26. 10.1001/jamacardio.2017.4293 29188294 PMC5833524

[2] Sweeting MJ Masconi KL Jones E Ulug P Glover MJ Michaels JA Analysis of clinical benefit, harms, and cost-effectiveness of screening women for abdominal aortic aneurysm. Lancet. (2018) 392:487–95. 10.1016/S0140-6736(18)31222-4 30057105 PMC6087711

[3] Stepien KL Bajdak-Rusinek K Fus-Kujawa A Kuczmik W Gawron K. Role of extracellular matrix and inflammation in abdominal aortic aneurysm. Int J Mol Sci. (2022) 23(19):11078. 10.3390/ijms231911078 36232377 PMC9569530

[4] Sakalihasan N Michel JB Katsargyris A Kuivaniemi H Defraigne JO Nchimi A Abdominal aortic aneurysms. Nat Rev Dis Primers. (2018) 4(1):34. 10.1038/s41572-018-0030-7 30337540

[5] Sorvillo N Cherpokova D Martinod K Wagner DD. Extracellular DNA NET-works with dire consequences for health. Circ Res. (2019) 125(4):470–88. 10.1161/CIRCRESAHA.119.314581 31518165 PMC6746252

[6] Villahoz S Yunes-Leites PS Méndez-Barbero N Urso K Bonzon-Kulichenko E Ortega S Conditional deletion of Rcan1 predisposes to hypertension-mediated intramural hematoma and subsequent aneurysm and aortic rupture. Nat Commun. (2018) 9:4795. 10.1038/s41467-018-07071-7 30442942 PMC6237779

[7] Chen P-Y Qin L Li G Malagon-Lopez J Wang Z Bergaya S Smooth muscle cell reprogramming in aortic aneurysms. Cell Stem Cell. (2020) 26:542–557.e11. 10.1016/j.stem.2020.02.013 32243809 PMC7182079

[8] Lee JS Park SC Kim SD. The relation between metabolic syndrome and aspects of abdominal aortic aneurysm. Asian J Surg. (2022) 45(1):307–14. 10.1016/j.asjsur.2021.05.046 34148751

[9] Menges AL D Oria M Zimmermann A Dueppers P. Ruptured abdominal aorto-iliac aneurysms: diagnosis, treatment, abdominal compartment syndrome, and role of simulation-based training. Semin Vasc Surg. (2023) 36(2):163–73. 10.1053/j.semvascsurg.2023.03.002 37330231

[10] Schanzer A Messina LM Ghosh K Simons JP Robinson WP Aiello FA Follow-up compliance after endovascular abdominal aortic aneurysm repair in medicare beneficiaries. J Vasc Surg. (2015) 61:16–22.e1. 10.1016/j.jvs.2014.06.006 25441010 PMC4276501

[11] Giese MA Hind LE Huttenlocher A. Neutrophil plasticity in the tumor microenvironment. Blood. (2019) 133:2159–67. 10.1182/blood-2018-11-844548 30898857 PMC6524564

[12] Yang Y Hozawa A Kogure M Narita A Hirata T Nakamura T Dietary inflammatory Index positively associated with high-sensitivity C-reactive protein level in Japanese from NIPPON DATA2010. Journal of Epidemiology. (2020) 30:98–107. 10.2188/jea.JE20180156 30745493 PMC6949183

[13] Baumer Y Dey AK Gutierrez-Huerta CA Khalil NO Sekine Y Sanda GE Hyperlipidaemia and IFNgamma/TNFalpha synergism are associated with cholesterol crystal formation in endothelial cells partly through modulation of lysosomal pH and cholesterol homeostasis. eBioMedicine. (2020) 59:102876. 10.1016/j.ebiom.2020.102876 32646751 PMC7502673

[14] Jin Y Li J Chen J Shao M Zhang R Liang Y Tissue-specific autoantibodies improve diagnosis of primary Sjögren’s syndrome in the early stage and indicate localized salivary injury. J Immunol Res. (2019) 2019:1–8. 10.1155/2019/3642937 31205955 PMC6530237

[15] Wilson CH Kumar S. Caspases in metabolic disease and their therapeutic potential. Cell Death Differ. (2018) 25:1010–24. 10.1038/s41418-018-0111-x 29743560 PMC5988802

[16] Bradley NA Walter A Wilson A Siddiqui T Roxburgh CSD McMillan DC The prognostic value of preoperative systemic inflammation-based scoring in patients undergoing endovascular repair of abdominal aortic aneurysm. J Vasc Surg. (2023) 78(2):362–369.e2. 10.1016/j.jvs.2023.04.018 37086821

[17] Shalaby SY Foster TR Hall MR Brownson KE Vasilas P Federman DG Systemic inflammatory disease and its association with type II endoleak and late interventions after endovascular aneurysm repair. JAMA Surg. (2016) 151(2):147–53. 10.1001/jamasurg.2015.3219 26501863

[18] Chai X Chen Y Li Y Chi J Guo S. Lower geriatric nutritional risk index is associated with a higher risk of all-cause mortality in patients with chronic obstructive pulmonary disease: a cohort study from the national health and nutrition examination survey 2013–2018. BMJ Open Resp Res. (2023) 10:e001518. 10.1136/bmjresp-2022-001518 37474197 PMC10357806

[19] Sun Y-H Hu N-Q Huang X-Y Liu Z-X Li Q-Y Li Q-L Central and peripheral blood pressures in relation to the triglyceride-glucose index in a Chinese population. Cardiovasc Diabetol. (2024) 23:3. 10.1186/s12933-023-02068-z 38172813 PMC10765647

[20] Roth GA Mensah GA Johnson CO Addolorato G Ammirati E Baddour LM Global burden of cardiovascular diseases and risk factors, 1990–2019. J Am Coll Cardiol. (2020) 76:2982–3021. 10.1016/j.jacc.2020.11.010 33309175 PMC7755038

[21] Mahemuti N Jing X Zhang N Liu C Li C Cui Z Association between systemic immunity-inflammation Index and hyperlipidemia: a population-based study from the NHANES (2015–2020). Nutrients. (2023) 15:1177. 10.3390/nu15051177 36904176 PMC10004774

[22] Cheng W Bu X Xu C Wen G Kong F Pan H Higher systemic immune-inflammation index and systemic inflammation response index levels are associated with stroke prevalence in the asthmatic population: a cross-sectional analysis of the NHANES 1999–2018. Front Immunol. (2023) 14:1191130. 10.3389/fimmu.2023.1191130 37600830 PMC10436559

[23] Esparham A Shoar S Mehri A Modukuru VR. Bariatric surgery and cardiovascular disease risk in patients with pulmonary hypertension: a propensity score matched analysis of US national inpatient sample. Obes Surg. (2023) 33:3230–6. 10.1007/s11695-023-06799-6 37639208

[24] Windsor MT Bailey TG Perissiou M Greaves K Jha P Leicht AS Acute inflammatory responses to exercise in patients with abdominal aortic aneurysm. Med Sci Sports Exerc. (2018) 50:649–58. 10.1249/MSS.0000000000001501 29210916

[25] Golledge J Clancy P Yeap BB Hankey GJ Norman PE. Increased serum angiopoietin-2 is associated with abdominal aortic aneurysm prevalence and cardiovascular mortality in older men. Int J Cardiol. (2013) 167:1159–63. 10.1016/j.ijcard.2012.03.120 22483260

[26] King AH Schmaier AH Harth KC Kumins NH Wong VL Zidar DA Elevated neutrophil-lymphocyte ratio predicts mortality following elective endovascular aneurysm repair. J Vasc Surg. (2020) 72(1):129–37. 10.1016/j.jvs.2019.10.058 32037083

[27] Muehling BM Ortlieb L Oberhuber A Orend KH. Fast track management reduces the systemic inflammatory response and organ failure following elective infrarenal aortic aneurysm repair. Interact Cardiovasc Thorac Surg. (2011) 12:784–8. 10.1510/icvts.2010.262337 21343153

[28] Yuan Z Lu Y Wei J Wu J Yang J Cai Z. Abdominal aortic aneurysm: roles of inflammatory cells. Front Immunol. (2021) 11:609161. 10.3389/fimmu.2020.609161 33613530 PMC7886696

[29] Wagenhäuser MU Mulorz J Krott KJ Bosbach A Feige T Rhee YH Crosstalk of platelets with macrophages and fibroblasts aggravates inflammation, aortic wall stiffening, and osteopontin release in abdominal aortic aneurysm. Cardiovasc Res. (2024) 120(4):417–32. 10.1093/cvr/cvad168 37976180

[30] Filiberto AC Spinosa MD Elder CT Su G Leroy V Ladd Z Endothelial pannexin-1 channels modulate macrophage and smooth muscle cell activation in abdominal aortic aneurysm formation. Nat Commun. (2022) 13(1):1521. 10.1038/s41467-022-29233-4 35315432 PMC8938517

[31] Lu S White JV Nwaneshiudu I Nwaneshiudu A Monos DS Solomides CC Human abdominal aortic aneurysm (AAA): evidence for an autoimmune antigen-driven disease. Autoimmun Rev. (2022) 21(10):103164. 10.1016/j.autrev.2022.103164 35926768

[32] Irimia A Sarkar A Stanfield RL Wilson IA. Crystallographic identification of lipid as an integral component of the epitope of HIV broadly neutralizing antibody 4E10. Immunity. (2016) 44:21–31. 10.1016/j.immuni.2015.12.001 26777395 PMC4720917

[33] Jolly SS Cairns JA Yusuf S Rokoss MJ Gao P Meeks B Outcomes after thrombus aspiration for ST elevation myocardial infarction: 1-year follow-up of the prospective randomised TOTAL trial. Lancet. (2016) 387:127–35. 10.1016/S0140-6736(15)00448-1 26474811 PMC5007127

[34] Ha CWY Martin A Sepich-Poore GD Shi B Wang Y Gouin K Translocation of viable gut Microbiota to mesenteric adipose drives formation of creeping fat in humans. Cell. (2020) 183:666–683.e17. 10.1016/j.cell.2020.09.009 32991841 PMC7521382

[35] Zhu F Willette-Brown J Song N-Y Lomada D Song Y Xue L Autoreactive T cells and chronic fungal infection drive esophageal carcinogenesis. Cell Host Microbe. (2017) 21:478–493.e7. 10.1016/j.chom.2017.03.006 28407484 PMC5868740

[36] Lobry T Miller R Nevo N Rocca CJ Zhang J Catz SD Interaction between galectin-3 and cystinosin uncovers a pathogenic role of inflammation in kidney involvement of cystinosis. Kidney Int. (2019) 96(2):350–62. 10.1016/j.kint.2019.01.029 30928021 PMC7269416

[37] Popescu NI Silasi R Keshari RS Girton A Burgett T Zeerleder SS Peptidoglycan induces disseminated intravascular coagulation in baboons through activation of both coagulation pathways. Blood. (2018) 132(8):849–60. 10.1182/blood-2017-10-813618 29921614 PMC6107880

[38] Park S-Y Matte A Jung Y Ryu J Anand WB Han E-Y Pathologic angiogenesis in the bone marrow of humanized sickle cell mice is reversed by blood transfusion. Blood. (2020) 135(23):2071–84. 10.1182/blood.2019002227 31990287 PMC7273832

[39] Oliveira BDD Xu K Shen TH Callahan M Kiryluk K D’Agati VD Molecular nephrology: types of acute tubular injury. Nat Rev Nephrol. (2019) 15(10):599–612. 10.1038/s41581-019-0184-x 31439924 PMC7303545

[40] Khoury HJ Gambacorti-Passerini C. Practical management of toxicities associated with bosutinib in patients with Philadelphia chromosome-positive chronic myeloid leukemia. Ann Oncol. (2018) 29(3):578–87. 10.1093/annonc/mdy019 29385394 PMC5888919

